# Gene expression profiles provide insights into the survival strategies in deep-sea mussel (*Bathymodiolus platifrons*) of different developmental stages

**DOI:** 10.1186/s12864-022-08505-9

**Published:** 2022-04-19

**Authors:** Junrou Huang, Peilin Huang, Jianguo Lu, Nengyou Wu, Genmei Lin, Xilin Zhang, Hong Cao, Wei Geng, Bin Zhai, Cuiling Xu, Zhilei Sun

**Affiliations:** 1 School of Marine Sciences, Sun Yat-sen University, Zhuhai, 519082 China; 2 Southern Laboratory of Ocean Science and Engineering (Guangdong, Zhuhai), Zhuhai, 519000 Guangdong China; 3 Guangdong Provincial Key Laboratory of Marine Resources and Coastal Engineering, Guangzhou, 510275 Guangdong China; 4 Pearl River Estuary Marine Ecosystem Research Station, Ministry of Education, Zhuhai, 519000 China; 5 Key Laboratory of Gas Hydrate, Ministry of Natural Resources, Institute of Marine Geology, China Geological Survey, Qingdao, 266071 China; 6 Laboratory for Mineral Resources, Qingdao Pilot National Laboratory for Marine Sciences and Technology, Qingdao, 266071 China

**Keywords:** Deep-sea mussel, Cold seep, Body size, Development, Adaptation, Symbiont regulation

## Abstract

**Background:**

Deep-sea mussels living in the cold seeps with enormous biomass act as the primary consumers. They are well adapted to the extreme environment where light is absent, and hydrogen sulfide, methane, and other hydrocarbon-rich fluid seepage occur. Despite previous studies on diversity, role, evolution, and symbiosis, the changing adaptation patterns during different developmental stages of the deep-sea mussels remain largely unknown.

**Results:**

The deep-sea mussels (*Bathymodiolus platifrons*) of two developmental stages were collected from the cold seep during the ocean voyage. The gills, mantles, and adductor muscles of these mussels were used for the Illumina sequencing. A total of 135 Gb data were obtained, and subsequently, 46,376 unigenes were generated using de-novo assembly strategy. According to the gene expression analysis, amounts of genes were most actively expressed in the gills, especially genes involved in environmental information processing. Genes encoding Toll-like receptors and sulfate transporters were up-regulated in gills, indicating that the gill acts as both intermedium and protective screen in the deep-sea mussel. Lysosomal enzymes and solute carrier responsible for nutrients absorption were up-regulated in the older mussel, while genes related to toxin resistance and autophagy were up-regulated in the younger one, suggesting that the older mussel might be in a vigorous stage while the younger mussel was still paying efforts in survival and adaptation.

**Conclusions:**

In general, our study suggested that the adaptation capacity might be formed gradually during the development of deep-sea mussels, in which the gill and the symbionts play essential roles.

**Supplementary Information:**

The online version contains supplementary material available at 10.1186/s12864-022-08505-9.

## Background

The deep-sea habitats, including cold seeps and hydrothermal vents, were established based on the unique light-independent chemosynthetic communities. Cold seeps were usually developed on the sloping seafloor of active and passive continental margins and in the subduction zones. They were flooded with hydrocarbons, mainly methane, hydrogen sulfide, fine-grained sediments, and also a certain amount of heavy metals. The water temperature usually ranged from less than 2 °C to about 8 °C [1], which is not so cold but slightly warmer than the surrounding water, while the water pressure was relatively high. Tubular worms, bivalves, annelids, gastropods, sea stars, and sea urchins are common primary consumers in the cold seep ecosystems, while the community structures are often closely related to the distribution of the seepages and the dominant populations are also determined by other physicochemical factors such as fluid flow rates.

Species diversity in cold seeps is relatively low, as they are mostly dominated by only one or two species with very high biomass. Mussels are one of the most successful dominant species in cold seeps, especially *Bathymodiolus platifrons*, *B. childressi*, *B.azoricus*, and other *B.* sp. found in dense patches around seepages, forming mussel beds. *B. platifrons* was first described by Hashimoto and Okutani in 1994. It became one of the most common species reported in both cold seeps and hydrothermal vents in the South China Sea [2], East China Sea [3], Sagami Bay, and Okinawa Trough [4]. To survive in such extreme physical and ecological environments, special physiological structures and metabolic mechanisms were formed with adaptive evolution and natural selection. The excellent survival strategy, cooperating with bacteria, was used by the Bathymodiolin species. Bacteria like methane-oxidizing bacteria, sulfur-oxidizing bacteria, and other symbiotic chemoautotrophic bacteria provide nutrients and energy sources for mussels [5]. There could be one or multiple species of bacteria living inside the specialized gill epithelial cells (i.e., bacteriocytes) of the mussels or on other epithelial tissues. In addition, the capacity of filter-feeding was retained after the establishment of endosymbiosis [6–8].

Diets of bathymodiolins were likely to change from heterotrophy to mixotrophy and then to chemosymbiosis in their early life. This transition may be closely related to the colonization and proliferation of symbiotic bacteria during the life history [9]. Symbionts are generally acquired in the juvenile stage of bathymodiolins, during which most of the epithelial tissues were infected. With increasing age as well as body size, the number and density of symbionts in the gill bacteriocytes grow, while bacteria on other tissues would gradually be removed [10].

Regulatory mechanisms for establishing and adjusting the symbionts remain unclear, during which mussels had to distinguish pathogens from symbionts and ensure the beneficial but not excessive proliferation of the symbionts. On the other hand, the number and density of symbionts vary considerably between developmental stages and have essential effects on important life processes, such as material and energy metabolism. Many of the previous studies have focused on the mechanisms of host-symbiont interactions. However, changes that occurred in different developmental stages of mussels were rarely got noticed. We predicted that the number, density, and composition of symbionts varies along with the development of mussels and may have essential effects on their immune response and nutrient acquisition. At the same time, mussels would also pay efforts to maintain the stability of symbiotic population and composition. Here, we utilized different tissues of mussels of different developmental stages for comparative transcriptome analysis, trying to figure out the questions above on the expression level.

## Results

### Species identification

**Fig. 1:**
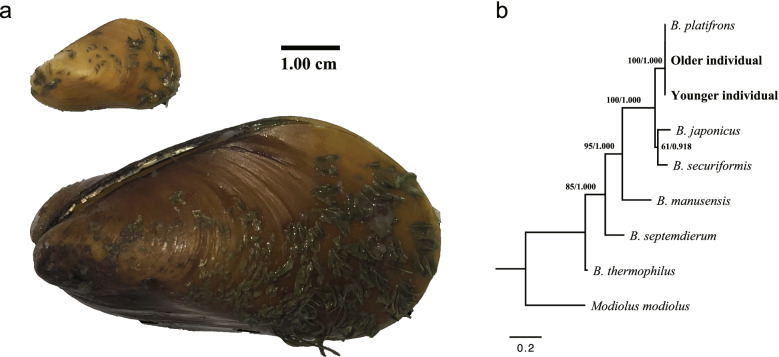
Deep-sea mussel *Bathymodiolus platifrons* collected from the cold-seep; **a** Comparison of the size of mussels. **b** Multi-gene tree constructed with COI and *atp6*

The number of positions of the original alignments of *atp6* and COI was 923 and 723, and 1235 sites were remained after concatenating the two trimmed alignments. Trees constructed with either Maximum Likelihood or Bayesian estimation using either the best-fit model HKY + G or GTR + I + G were always topological congruent, in which *Bathymodiolus platifrons* and the two individuals used in our study form a clade, indicating a close relationship (Fig. 1b). Besides, the two individuals share a much higher sequence identity with *B. platifrons* than with other species, and the genetic distances were also smaller (Additional file 2 Table S1). Mussels used in our study were finally identified as *B. platifrons*.

### Summary of transcriptome data

To minimize the data variance and ensure reliability, 18 libraries were constructed and sequenced independently for three replicates of each tissue. One hundred thirty-six Gb raw data obtained from Illumina sequencing, and 130 Gb clean data, more than 6.13 Gb for each sample, were retained for subsequent analysis (Additional file 2 Table S2, S3). Forty-six thousand three hundred seventy-six unigenes, as well as 64,672 transcripts, were generated after assembly, and the N50 length of unigenes was 1389 (Additional file 2 Table S4, Additional file 1 Fig. S1). A total of 17,150 of 46,376 unigenes (37%) were annotated by six databases, while 8196 to 14,755 by each (Additional file 2 Table S5).

### Global view of different gene expression patterns

**Fig. 2:**
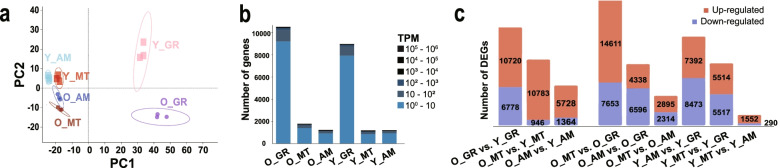
The overview of gene expression pattern in all samples; **a** Clustering of the 18 samples using principal component analysis. **b** The number of expressed genes in each tissue. **c** Number of DEGs in inter-individual as well as inter-tissue comparison groups. O_GR, O_MT, O_AM: the gill, mantle, adductor muscle of the older individual; Y_GR, Y_MT, Y_AM: the gill, mantle, adductor muscle of the younger individual

Distinct gene expression patterns were shown in the gills of both the older and the younger individuals. The number of expressed genes in gills was significantly higher than in other tissues, as up to ten thousand genes were expressed in gill, while much fewer genes were expressed in the mantle or adductor muscle (Fig. 2b). Five hundred sixty-nine genes were found expressed in all samples. The peak of the distribution of genes expressed in gills was also much higher than that in other tissue. Furthermore, the universal expression levels were slightly higher in tissues of the older individual (Additional file 1 Fig. S2).

To provide a global view of the divergence of gene expression profiles and investigate whether the transcriptomes are specifically correlated with tissues and the size of mussels (Table 1), datasets were subjected to principal component analysis (PCA), hierarchical clustering, and calculation of distance correlation. Distance between individuals was relatively high comparing with that between tissues (Additional file 1 Fig. S3). Samples of the same tissues were well clustered, and samples of gills were clustered into a separate branch (Additional file 1 Fig. S4). Correlations among samples were better revealed in the PCA plot (Fig. 2a), as PC2 separated the individuals into different sizes and PC1 sufficiently distinguished gills from other tissues. Samples of the mantle and adductor muscle were also explicitly clustered, while the relatively small distance between them was corresponding to the more similar number or expression patterns of genes.

**Table 1:** Comparision of size and weight of the individual

	Length (mm)	Width (mm)	Height (mm)	Wet weight (g)	Height / Length	Weight / Length
Older individual	65.0	26.0	33.0	25.40	0.51	0.40
Younger individual	25.0	9.0	15.6	1.97	0.62	0.36

### Differential expression analysis between mussels

To figure out the important life process specific to developmental stages, comparative groups of the same tissues between individuals were generated respectively. Three comparative groups were generated: GR (younger-gill vs. Older-gill), MT (younger-mantle vs. older-mantle), AM (younger-adductor muscle vs. older-adductor muscle).

**Fig. 3:**
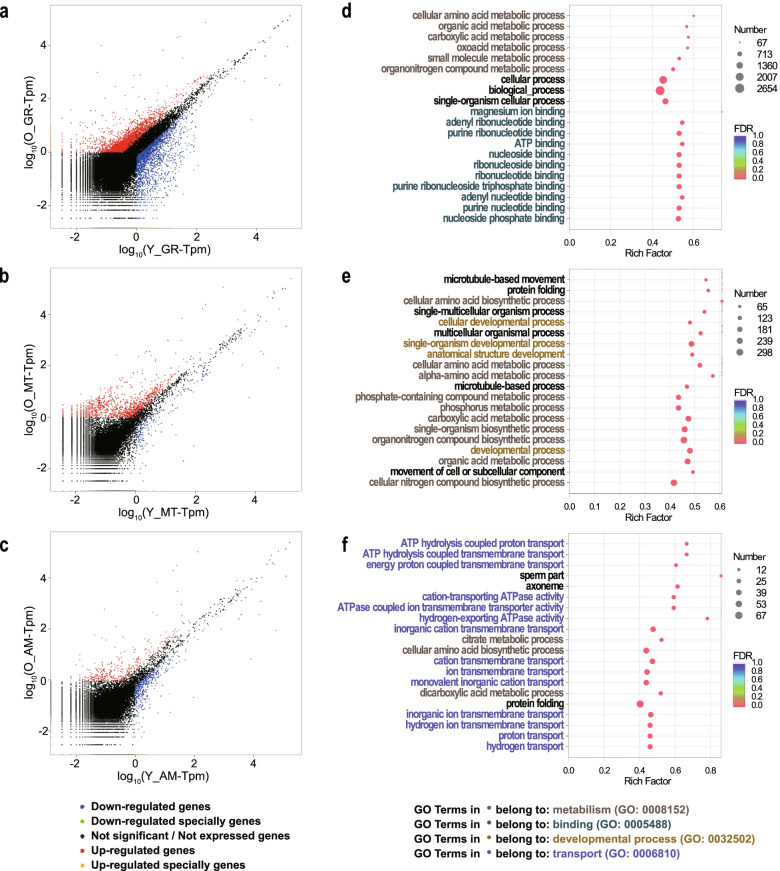
The scatter plot of gene expression and GO enrichment results; **a**-**c** Scatter plot of the genes in each comparative group. Red spots were genes significantly up-regulated in the older individual, and blue spots were genes significantly down-regulated. **d**-**f** The most enriched 20 GO terms in each comparison group. GO terms painted with blue, green, orange, or red were involved in specific ancestor GO terms. O_GR, O_MT, O_AM: the gill, mantle, adductor muscle of the older individual; Y_GR, Y_MT, Y_AM: the gill, mantle, adductor muscle of the younger individual

The Venn diagrams show the number of common and specific genes expressed within comparative groups (Additional file 1 Fig. S5). Relatively more genes were specifically expressed in the older individual in all tissues, and shared genes were always the largest portion. The number of differentially expressed genes (DEGs) decreased sequentially in GR, MT, and AM (Fig. 3a-c). More genes were up-regulated in the older individual within all comparative groups, and disequilibrium was shown in the ratio between up- and down-regulated genes in MT or AM.

Up to 39,665 common genes were shared by gills of the two individuals, while no more than two thousand specific genes in each, showing the most significant contrast in all comparative groups (Additional file 1 Fig. S5, Additional file 2 Table S5). However, the number of DEGs in GR was the highest, and the up-down ratio was relatively balanced, suggesting that most of the genes in gills may keep functioning throughout the growth of mussels, but serve for biological processes specific to developmental stages.

In order to investigate the shift of the central biological process along with growth and development, annotation and enrichment of both gene function and biological pathway were carried out (Additional file 2 Table S6). COG categories and the GO terms (Level 2) to which most DEGs were annotated showed that metabolism and cellular processes such as translation and transportation varied between individuals in all tissues (Additional file 1 Fig. S6, S7), revealing different development statuses.

GO enrichment was performed in DEGs of each comparative group, and in conclusion, 94, 343 and 246 GO terms were enriched (p.adjust < 0.01) in GR, MT, and AM, respectively. The most significantly enriched GO terms of each comparative group were shown in Fig. 3d-f. GO terms involved in metabolism were specifically enriched in all comparative groups, especially in MT, suggesting an overall metabolic level difference between individuals. DEGs of GR were specifically enriched in substance binding, especially nucleotide and ribonucleotide binding, suggesting that the individuals may differ in energy storage and transfer or pathogens recognition. The greatest difference in AM was various sorts of transport, such as the proton, hydrogen, inorganic cation, or inorganic ion, suggesting a difference in energy transformation or regulation of osmotic pressure.

KEGG enrichment was also performed in DEGs of all comparative groups, but the up- and down-regulated DEGs were enriched separately in order to reveal the biological process specifically up-regulated in each tissue. Pathways with p.adjust < 0.05 were considered to be significantly enriched. Those related to metabolism or genetic information processing were specifically enriched in all tissues of the older individual, while pathways specifically enriched in the younger one were mainly related to resistance or response to drugs, pathogens, or disease (Table 2, Additional file 1 Fig. S8, S9, S10).

**Table 2:** The most enriched 10 KEGG pathways in either up- or down-regulated genes in each tissue in the comparison between individuals

	Genes up-regulated in the older individual			Genes up-regulated in the younger individual		
	Map ID	Pathway description	p.adjust	Map ID	Pathway description	p.adjust
**Gill**	map03010	Ribosome ^a^[M]	7.2E-04	map05200	Pathways in cancer ^a^	7.7E-05
	map04141	Protein processing in endoplasmic reticulum ^a^[M]	1.5E-02	map05145	Toxoplasmosis ^a^[I]	7.7E-05
	map00230	Purine metabolism ^a^[M]	1.5E-02	map05206	MicroRNAs in cancer ^a^	7.7E-05
	map00240	Pyrimidine metabolism ^a^[M]	1.5E-02	map04510	Focal adhesion ^a^	8.3E-05
	map00260	Glycine, serine and threonine metabolism ^a^[M]	2.3E-02	map04064	NF-kappa B signaling pathway ^a^	8.3E-05
	map04730	Long-term depression ^a^	2.6E-02	map05222	Small cell lung cancer ^a^	8.3E-05
	map04341	Hedgehog signaling pathway ^a^	2.8E-02	map04668	TNF signaling pathway ^a^[I]	8.3E-05
	map00290	Valine, leucine and isoleucine biosynthesis ^a^[M]	2.8E-02	map05164	Influenza A ^a^[I]	9.4E-05
	map00300	Lysine biosynthesis ^a^[M]	2.8E-02	map04621	NOD-like receptor signaling pathway ^a^[I]	1.0E-04
	map04742	Taste transduction	6.2E-02	map04215	Apoptosis - multiple species ^a^	2.1E-04
**Mantle**	map04141	Protein processing in endoplasmic reticulum ^a^[M]	1.6E-04	map05145	Toxoplasmosis ^a^[I]	1.3E-04
	map00230	Purine metabolism ^a^[M]	1.7E-03	map04621	NOD-like receptor signaling pathway ^a^[I]	1.3E-04
	map04742	Taste transduction ^a^	1.7E-03	map04624	Toll and Imd signaling pathway ^a^[I]	4.1E-04
	map04730	Long-term depression ^a^	4.3E-03	map04668	TNF signaling pathway ^a^	5.8E-04
	map04341	Hedgehog signaling pathway ^a^	1.2E-02	map04215	Apoptosis - multiple species ^a^	1.2E-03
	map03010	Ribosome ^a^[M]	1.2E-02	map05222	Small cell lung cancer ^a^	4.7E-03
	map00260	Glycine, serine and threonine metabolism ^a^[M]	1.2E-02	map05134	Legionellosis ^a^[I]	6.4E-03
	map05110	*Vibrio cholerae* infection ^a^[I]	3.0E-02	map05152	Tuberculosis ^a^[I]	2.1E-02
	map00240	Pyrimidine metabolism ^a^[M]	3.1E-02	map04640	Hematopoietic cell lineage ^a^[I]	2.1E-02
	map00290	Valine, leucine and isoleucine biosynthesis ^a^[M]	3.2E-02	map01524	Platinum drug resistance ^a^[I]	2.6E-02
**Adductor muscle**	map03010	Ribosome ^a^[M]	9.3E-14	map05100	Bacterial invasion of epithelial cells ^a^[I]	2.2E-07
	map03050	Proteasome ^a^[M]	3.9E-05	map04810	Regulation of actin cytoskeleton ^a^	9.6E-07
	map04141	Protein processing in endoplasmic reticulum ^a^[M]	9.8E-03	map05131	Shigellosis ^a^	3.3E-06
	map00020	Citrate cycle (TCA cycle)	5.1E-02	map05120	Epithelial cell signaling in *Helicobacter pylori* infection ^a^[I]	1.5E-04
	map00740	Riboflavin metabolism	5.1E-02	map05130	Pathogenic *Escherichia coli* infection ^a^[I]	1.8E-04
	map03060	Protein export	5.1E-02	map04666	Fc gamma R-mediated phagocytosis ^a^[I]	1.8E-04
	map05150	*Staphylococcus aureus* infection	5.1E-02	map00190	Oxidative phosphorylation ^a^	2.6E-04
	map00500	Starch and sucrose metabolism	5.1E-02	map04670	Leukocyte transendothelial migration ^a^[I]	5.0E-04
	map04721	Synaptic vesicle cycle	5.6E-02	map04530	Tight junction ^a^	8.0E-04
	map05110	*Vibrio cholerae* infection	6.4E-02	map04932	Non-alcoholic fatty liver disease (NAFLD) ^a^	1.6E-03

[M]: Pathways involved in metabolism or genetic information processing

[I]: Pathways involved in infectious diseases or immune system

^a ^Significantly enriched pathways

### Differential expression analysis between tissues

Gills, a nutritive organ, act as shields in mussels: directly exposed to the water and transfer enriched substances. To better understand the role of the gills and their potential mechanism in deep-sea mussels, we conducted a comparative transcriptome analysis between the three tissues: gill, which are in direct contact with seawater, the adductor muscle that hardly exchanges substances with the external environment, and the mantle between them.

**Fig. 4:**
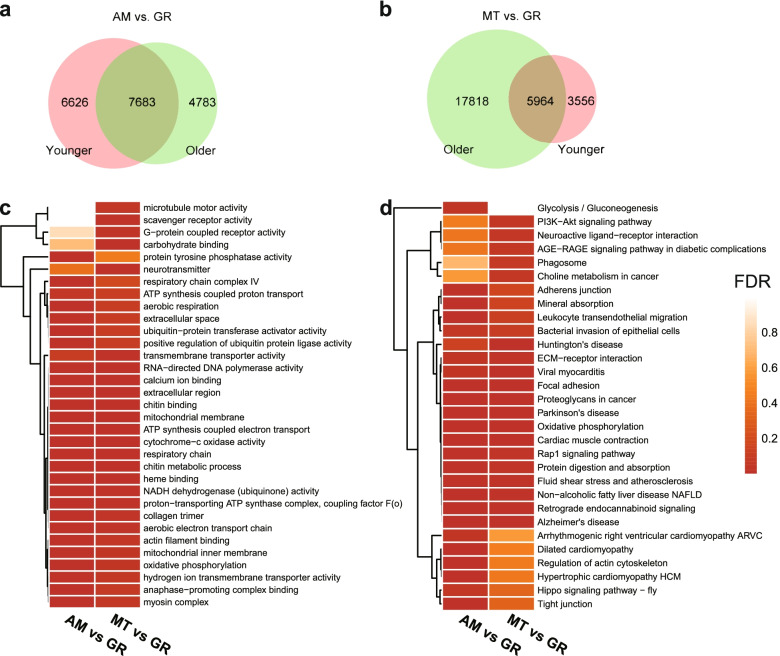
The DEGs when comparing the other two tissues (adductor muscle, AM; mantle, MT) with the gill (GR); **a** The number of DEGs. **b** The Venn diagrams of DEGs. The results of GO terms (**c**) and KEGG pathways (**d**) enrichment for the overlapping genes in (**a**) and (**b**). The colors in the heatmaps represent the value of FDR (false discovery rate). O_GR, O_MT, O_AM: the gill, mantle, adductor muscle of the older individual; Y_GR, Y_MT, Y_AM: the gill, mantle, adductor muscle of the younger individual

Comparing with the other two tissues, genes in the gills were found most actively expressed as inferred from the greater number of expressed genes with higher expression levels (Fig. 2b-c). The expression differences between adductor muscle and mantle were not significant (Fig. 2c), indicating that the environment is a fundamental influence factor for gene expression. The expression activity may positively correlate with the degree of exposure to the external environment. The largest number of 22,264 DEGs were identified when comparing the mantle to the gill from the older individual (Fig. 4b). However, 15,865 DEGs between adductor muscle and gill were found to be the maximum number in the younger one. When comparing the other two tissues with the gill (Fig. 4a-b), a large number of unigenes (7683) were differentially expressed between the adductor muscle and the gill both in the younger (7683/14,309, 53.7%) and older individuals (7683/12,446, 61.7%). There were fewer unigenes found differentially expressed between the mantle and the gill, both in the younger (5964/9520, 62.6%) and older individuals (5964/23,782, 25.1%). We constructed gene sets containing 7683 and 5964 genes, respectively (i.e., the overlapping genes in Fig. 4a-b). Then, functional enrichment analysis for these genes was performed. Interestingly, the GO and KEGG enrichment results for the two different gene sets were quite close (Fig. 4c-d). They were enriched with 33 GO terms such as cytochrome-c oxidase activity, ATP synthesis coupled electron transport, transmembrane transporter activity, and positive regulation of ubiquitin protein ligase activity (Fig. 4c) as well as 30 KEGG pathways such as mineral absorption, bacterial invasion of epithelial cells, and phagosome (Fig. 4d).

### Selection and validation of candidate genes

**Fig. 5:**
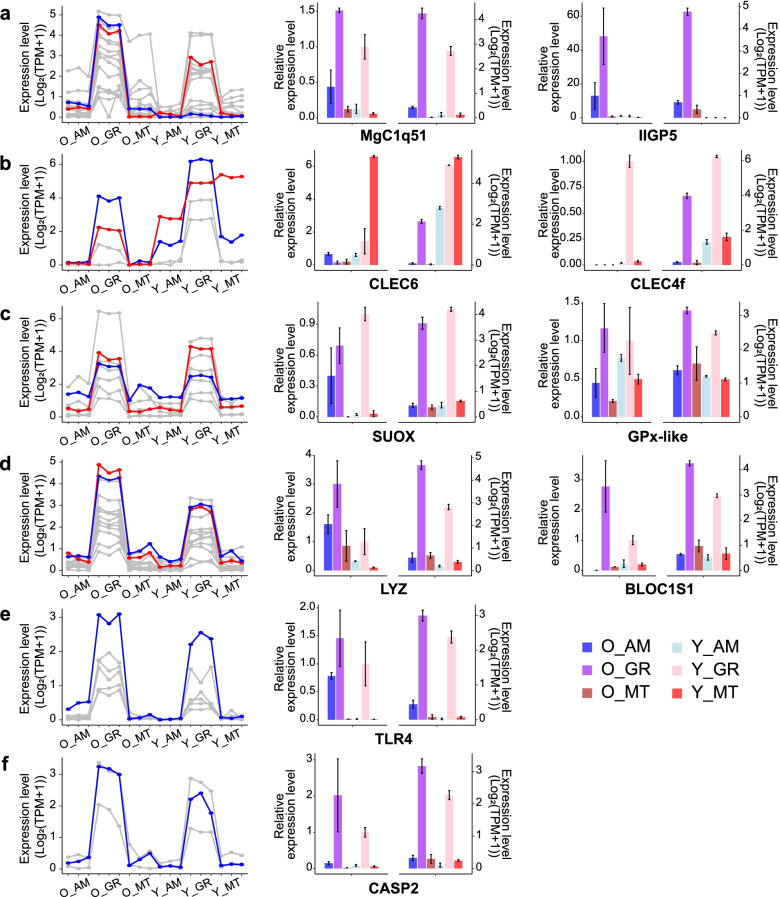
qRT-PCR results of the ten validated genes; The relative expression levels of qRT-PCR results were shown by bar plot on the left side, and the corresponding expression levels in the transcriptome were shown by bar plot on the right side for each gene. Several categories of genes on which were concentrated in the study were shown in the line charts specifically, and the validated genes were highlighted in either red or blue. O_GR, O_MT, O_AM: the gill, mantle, adductor muscle of the older individual; Y_GR, Y_MT, Y_AM: the gill, mantle, adductor muscle of the younger individual

According to the differential expression analysis, both between mussels and between tissues, and the results of GO and KEGG enrichment, a series of genes were found potentially related to the underlying mechanisms of symbionts regulation and the survival strategies of these deep-sea mussels. Genes involved in five categories, including immune response, stress response, lysosome formation and function, Toll-like receptors, and apoptosis, were considered vital roles in metabolism, symbiont recognition, symbiont population control, and deep-sea environment adaptation (Fig. 5, Additional file 2 Table S7). To verify the reliability of gene expression patterns calculated with the transcriptome data, ten genes were randomly selected from each category and used for qRT-PCR validation. The relative expression level of each gene in qRT-PCR was similar to the expression level in transcriptome data, suggesting that the gene expression profile generated from the RNA-seq data in our study was reliable to a certain extent.

## Discussion

The deep-sea cold seeps are mysterious regions with large biomass. Comparative analysis between mussels and their tissues provided us a global view of the fundamental and unique life process in different developmental stages and the specific roles of tissues in the face of the surrounding deep-sea water rich in sulfide and heavy metals. We found that the younger and the older individuals showed a significant difference in metabolism and responsiveness, as the expression levels of genes related to metabolism, binding, transportation, and the immune response showed relatively higher differential expressions. At the same time, it was also supported by the result of function and pathway enrichment. In addition, the gills, uniquely compared to the other two tissues, were found to serve as the “peripheral defense” line in mussels. Our results show consistency with previous research results.

### Dynamics of the host-symbiont interactions and regulation

The density and abundance of symbionts increase along with the host size, during which the immune system, the lysosomal system, and the ubiquitin–proteasome system may play important roles in the recognition, acquisition, and regulation of symbionts [11]. We focus on the different life activities of the host during development accompanied by symbiont establishment and maintenance.

It was reported that juvenile Bathymodiolins acquire nutrients through filter-feeding before the later development of gills [10]. However, the nutritional contribution of filter-feeding would decrease along with the growing sizes, as endosymbionts become the sources of the major nutrients [12]. The up-regulated lysosomal enzymes, lysozyme, lysosomal Pro-X carboxypeptidase, and lysosomal alpha-mannosidase may indicate that digestion of endosymbionts was more active in the older mussel, as mussels could directly digest endosymbionts for nutrients, which is called farming, in which the lysosomal enzymes were involved. Another way to acquire nutrients from endosymbionts was milking, in which the solute carriers (SLCs) play essential roles. SLCs were up-regulated in the gills of both mussels, among which the iron-regulated transporters SLC40, nucleotide sugar transporters SLC35, and putative sugar transporters SLC45 were specifically up-regulated in the older mussel [13–15]. The up-regulation of genes involved in both farming and milking may suggest a shift toward greater reliance on endosymbionts during the growth of the mussel. In addition, up-regulated genes in the gills of the younger mussel were enriched in several disease-related pathways, which may be a piece of evidence that the proportion of filter-feeding may gradually decrease during maturation, as gills would be constantly stimulated by the environmental microorganisms during filter-feeding. A higher level of filter-feeding has remained in the younger mussel [11].

Toll-like receptors (TLRs), C-type lectin receptors (CLRs), peptidoglycan recognition proteins (PGRPs) were membrane-bound pattern recognition receptors (PRRs), identifying pathogen-associated molecular patterns (PAMPs) on microbial pathogens [16]. TLRs, PGRPs, and CLR-containing C1q-like proteins were found up-regulated in the gill of the older mussel, as well as a series of other immune-related genes, such as MgC1q [17], members of the GTPase of the immune-associated protein (GIMAP), interferon-inducible GTPase5 (IIGP5) (Fig. 5, Additional file 2 Table S7). C-type lectin (CLECs) were important in recognizing pathogens and play important roles in homeostasis and antimicrobial immune responses [18]. Most of the members in the CLEC superfamily were found up-regulated in the gill of the older mussel, including CLEC1 and CLEC9. In contrast, CLEC4f and CLEC6 were found significantly up-regulated in the gill of the younger mussel, indicating that different CLECs recognizing specific ligands or having specific functions may play vital roles in different developmental stages specifically. The more active expression of the receptors in immune recognition may suggest more effective protection against infection in the gill of the older mussel, while the relatively inactive recognition receptors in the younger mussel may be one of the mechanisms to enable new symbionts colonization. In addition, the active defense system of the older individual suggested that environmental acquisition of symbionts may already be ceased, and the newly formed gill filaments acquire their symbionts from the older neighboring gill filaments [19].

Bacteria were engulfed into cells mainly through endocytosis. Caveolin-1, flotillin1, Ras-related protein 5 (RAB5), and early endosome antigen 1 (EEA1), which are major regulation factors in the clathrin-independent mechanisms of endocytosis, were up-regulated in the gill of the younger mussel. Ras-related C3 botulinum toxin substrate 1 (RAC1), p21-activated kinase 1 (PAK1), c-terminal-binding protein 1 (CTBP1), which are involved in the macropinocytosis-mediated endocytosis, were also up-regulated in the gill of the younger mussel. Comparing with clathrin-dependent endocytosis, clathrin-independent endocytosis is induced by pathogens or other specific signals, and macropinocytosis is the way through which bacteria and viruses could enter the cells [20]. Our data suggest that the younger mussel might be actively acquiring endosymbionts through macropinocytosis and clathrin-independent endocytosis.

The lysosomal enzymes, including lysozyme, lysosomal Pro-X carboxypeptidase, lysosomal alpha-mannosidase, and a panel of lysosomal proteases, were up-regulated in the gill of the older mussel. Mannose-6-phosphate receptors (M6PR) responsible for lysosomal enzymes sorting and transport, adaptor protein-3 (AP-3) responsible for lysosomal membrane proteins transport, vesicle-associated membrane protein 7 (VAMP7) mediating endosome-lysosome membrane fusion, vacuolar-type H^+^-ATPase (VHA) mediating the pH in the lysosomal lumen were also up-regulated in the gill of the older mussel as well as other genes in the lysosome pathway, which is vital in cellular protein degradation and removing invading bacteria and virus [21–24] (Fig. 5, Additional file 2 Table S7). Another protein degradation system, the ubiquitin-proteasome system (UPS), shows a minor difference, as either ubiquitin-activating enzyme or ubiquitin-conjugating enzyme expressed in similar levels, and different E3 ubiquitin-protein ligase families were respectively up-regulated in the gill of either the older or the younger mussel. Considering that the UPS is responsible for intracellular protein degradation while lysosome is responsible for cytoplasmic components, including endocytosed foreign materials [25], lysosome-mediated degradation may be the main pathway in not only pathogens removal but also symbiont abundance maintenance.

Despite the lysosome-mediated degradation, autophagy and apoptosis might also be essential killing mechanisms for mussels in pathogens removal, defense against infection, and symbiont abundance maintenance. Autophagy and apoptosis control the turnover of organelles and proteins within cells and cells within organisms, respectively [26]. Autophagy-related genes were specifically up-regulated in the gill of the younger mussel, including autophagy-related protein 2 and 16 (ATG2, ATG16), which are essential in autophagosome formation, as well as damage-regulated autophagy modulator 1 and 2 (DRAM1, DRAM2) which are contributing to autophagy induction [27–30]. Autophagy may probably be one of the killing mechanisms in the younger mussel, contributing to defense against infection, while the down-regulated autophagy may help to maintain endosymbionts in the older individual. On the other hand, the majority of pro-apoptosis caspases (caspase-2, − 3, − 6, − 7, − 8, − 9, − 10) involved in mediating cell death signaling transduction were up-regulated in the gill of the older mussel (Fig. 5, Additional file 2 Table S7).

A previous study had reported that more apoptotic cells were found in *B. thermophilus* with higher symbionts, suggesting that the mussel might control their symbiont population through the apoptotic process. Apoptosis may be another way of symbiont abundance maintenance for mussels.

Generally, the younger mussel actively acquired symbionts for nutrition sources while also paying efforts on pathogen defense, during which immune receptors and lysosome-related genes were down-regulated, and the autophagy-related genes were up-regulated. The older mussel with relatively sufficient symbionts had to control and maintain the symbiont abundancy, during which lysosome-related genes and apoptosis-related genes were up-regulated, and the autophagy was suppressed. Different gene expression profiles between the mussels revealed changes in the host activities in the host-symbiont interactions. Further time series transcriptome analysis may provide a more comprehensive description of the symbiont-regulating mechanisms during different developmental stages.

### Gills in stress response

The deep-sea mussels may respond to extreme environmental stress through specific potential regulatory mechanisms to increase the complexity of the system. This regulation is tissue-specific, i.e., gills are the primary performer of this process. There was a unique gene expression pattern in the gill compared to the adductor muscle and the mantle, which reminds us it is the “peripheral defense” line in mussels. Given the special role of the gill in the mussel, coupled with the relatively closer gene expression patterns of the other two tissues, led us to observe exactly what differences existed between the gill and the other two tissues. We have noticed that a large proportion of genes were constantly differentially expressed in both the younger and the older mussels (Fig. 4a,b). The high similarity of the enrichment analysis results for the overlapping DEGs in Fig. 4a and b suggested that we are most likely to find the key genes that shape the defense role of gills in the intersection of these two gene sets.

Cold seeps are such environments with high concentrations of hydrogen sulfide, which is usually lethal for most organisms [31]. *B. platifrons*, as an organism that can maintain high biomass in such an area, have gills that cable of producing ATP from sulfide [32, 33] to support the mechanism of adaptation. In previous studies of animals living in hydrothermal vents, sulfide binding proteins were present in the blood of *Riflia pachyptila* and *Calyptogena magnifica* to protect hemoglobin, which is highly sensitive to sulfide. However, such a mechanism was not found in mussels [34], suggesting that mussels should respond to the high hydrogen sulfide environment by increasing the expression of genes related to sulfur metabolism in the same way as the shrimps in the seeps and vents. Now, in the two gene sets we built, we found sulfite oxidase (SUOX) predominantly expressed in the gill mitochondria (Fig. 5c, Additional file 2 Table S8). The top fold change value was observed between the mantle and the gill from, respectively, the older and younger individual. This finding not only supports that gills can oxidize sulfite through mitochondrial enzyme independently of symbiont-containing tissues, which is consistent with the results of Wong et al. [5], but also that this oxidative capacity may be enhanced over time by comparing mussels of different ages. When hydrogen sulfide enters the organism, the initial sulfide detoxification process is the oxidation of sulfide to thiosulfate by sulfide oxidase [33, 35]. We detected thiosulfate sulfurtransferase (TST) and sulfate anion transporter 1 (SAT1) up-regulated in the gills (Fig. 5c, Additional file 2 Table S8). TST is known to functionating in mussels in the metabolism of sulfite [36], however, the function of SAT1 was characterized in mammalian renal cell lines [37] and is still unclear in mussel species. In order to fully understand this sulfide-based ATP production, more knowledge is needed about their function and underlying mechanism in mussels. Our research will serve well to lay the groundwork for this purpose.

Vent effluents are known to have high concentrations of heavy metals, including iron, manganese, lead, zinc, copper, cadmium, chromium, and silver. However, the gills and mantles of mussels from cold seeps have also been reported to be enriched with high-level metals [38]. As a widely distributed species in the seep and vent regions [39], mussels are undoubtedly very successful in tolerating heavy metal stresses. In previous studies, metal-binding proteins such as metallothioneins [40, 41] were found to be beneficial. Unfortunately, in our study, no genes encoding metallothioneins were found to be significantly overexpressed in the gills. After the exposure, metals trigger the occurrence of reactive oxygen species (ROS), which causes the up-regulation of antioxidant-related enzymatic genes such as superoxide dismutase (SOD), catalase (CAT), and glutathione peroxidase (GPX) [42]. We found that there were several such unigenes specifically expressed in the gills (Fig. 5c, Additional file 2 Table S8), including Mn-SOD, CuZn-SOD, and GPX genes, suggesting that, as other normal species do, the regulation of antioxidant-related genes are activated by mussels to prevent the damage of metals. In the current study, we noticed that more genes in gill tissue were annotated to the KEGG pathway of ABC transporters. It has been speculated that ATP-binding cassette (ABC) transport proteins participate in alleviating the accumulation and toxicity of Ag nanoparticles in hemocytes and gill cells of mussels [43], indicating ABC pump may play an important role in the protection capacity of gills. Another strategy to protect cells from heavy metals was found in the mussels living in hydrothermal vents, which is the storage and segregation of both essential and non-essential metals in insoluble states [44]. Thus, it is probably reasonable to suppose that, for cold-seep mussels, activation of gene expression associated with ABC transporters and lysosomal biogenesis (Fig. 5c, Additional file 2 Table S8) may similarly be the result of heavy metal detoxification, as mentioned by Wang et al. [45].

Our RNA-seq results provide possible investigational directions for the mechanisms underlying the detoxification (including sulfides and heavy metals) of deep-sea mussels. More intensive work needs to be done to fulfill the relevant fields.

## Conclusion

In this study, we elucidated the differences in resistance to harsh environments, regulation of symbionts population, and metabolism of the younger and older deep-sea mussels *B. platifrons* living in the cold-seep area. Our results indicated that the gills act as the “peripheral defense” line in the mussels, and along with the development of the mussels, more stable relationships with both environmental and internal microorganisms were established. The reliance on endosymbionts grew greater, which may benefit from the more effective structure and the population growth of the endosymbionts in the older mussels.

Because of the difficulty of *B. platifrons* sample collection under the extreme condition, only a few *B. platifrons* individuals were captured for our research. But, we can get more samples for further study in this area at our future deep-sea survey. Interestingly, our work provides preliminary insights into the relationship between deep-sea mussels and the symbionts as well as the cold seep environment based on the whole transcriptomic scale. Our results shed the light for understanding the correlation of gene expression patterns with the deep-sea mussels development.

## Materials and methods

### Sampling

Mussels in the study were collected using the remotely operated vehicle (ROV) FCV3000 from a cold seep site on the west slope of the Okinawa Trough at a water depth of 896 m in October 2018. The length, width, and thickness of the shell of each mussel were measured before treatments. Gills, mantles, and the adductor muscles were dissected and frozen in liquid nitrogen and then stored at − 80 °C.

### DNA isolation and species identification

Total genomic DNA of the adductor muscle was isolated using E.Z.N.A.® Tissue DNA Kit according to the methods described in the manual. Previously described primers BP_atp6F & BP_atp6R [46] and LCO1490 & HCO2198 [3] for *atp6* and COI specifically were used for sequence amplification with TaKaRa PCR Amplification Kit. Polymerase chain reactions (PCRs) were performed with the following program: 2 min initial denaturation at 94 °C, 35 cycles of 35 s denaturation at 94 °C, 35 s annealing at 50 °C, 30 s extension at 72 °C, and a final extension for 10 min at 72 °C. DNA sequencing utilizing first-generation sequencing techniques was performed by the TIANYI HUIYUAN company. The sequence of *atp6* and COI of *B*. sp. and *Gigantidas* sp. were downloaded from NCBI (National Center for Biotechnology Information, https://www.ncbi.nlm.nih.gov/), as well as *Modiolus modiolus*, which was used as the outgroup.

Sequence alignment was performed by MUSCLE [47] and the multiple alignment were trimmed by Gblocks [48] with -b4 = 5 (Minimum Length Of A Block) and -b5 = None (Allowed Gap Positions). The multiple alignment of the two genes were then concatenated. The best-fit model of nucleotide substitution for phylogenetic analysis were selected using ModelFinder [49]. Both Maximun Likelihood (ML) trees and Bayesian trees were generated with the concatenated dataset, by IQ-TREE (Bootstrap = 1000) [50] and MrBayes (Generations = 2 × 10^7^; Sampling Freq = 100; average standard deviation of split frequencies < 0.05) [51, 52] specifically. Pairwise sequence identity and pairwise genetic distance were also used for supporting species identification. Sequence identity was calculated with an in-house script. Genetic distances were calculated using the Jukes-Cantor method (JC) [53], Kimura two parameters (K2P) method [54], Tamura 3-parameter method (T92) [55], LogDet methods [56] and p-distance method as implemented in MEGAX [57].

### RNA isolation and sequencing

Total RNA of gills, mantles, and the adductor muscles were isolated separately using SV Total RNA Isolation System (Promega, USA) according to the methods described in the manual, assuring three replicates for each tissue. Completeness and concentration were quantified by both agarose gel electrophoresis and BioSpec-nano (Shimadzu, Tokyo, Japan). Pair-end RNA sequencing was performed on Illumina PE150 (Shanghai Majorbio Bio-pharm Technology Co., Ltd).

### Transcriptome analysis

Quality control was performed on the raw data using SeqPrep and Sickle. Adaptors and low-quality bases on the 3′ end were first trimmed, and then reads containing Ns or shorter than 30 bp were removed to generate clean reads. Clean reads of each tissue were assembled independently using Trinity [58] and received subsequent evaluation and optimization by TransRate [59] and CD-HIT [60]. The completeness of the non-redundant assemblies was evaluated using BUSCO [61]. Six databases, NCBI-NR, Swiss-Prot [62], Pfam [63], COG [64], GO [65] and KEGG [66], were used in the functional and pathway annotation of both unigenes and transcripts specifically by DIAMOND [67], HMMER3 [68], BLAST2GO [69] and KOBAS [70].

Using the assembly as the reference sequence, the expression level of both genes and transcripts of each dataset were calculated by mapping clean reads to the reference with bowtie [71]. For read counts normalization, TPM (Transcripts Per Million reads) were calculated by RSEM [72]. The PCA analysis was performed with the TPM matrix using the built-in R function procomp() (scale = F, center = T), and PC1 (72.23%) and PC2 (12.24%) with the highest variation were then selected for PCA plotting. Hierachical clustering of the samples was based on Euclidean distance, using average linkage clustering method. Pearson correlation coefficients between samples were also calculated and presented as the correlation heatmap.

Differential expression analyses were performed by DESeq2 (R package) [73] between tissues or mussels. At the same time, if the |log_2_FC| (absolute value of the log fold change) of the expression level of either a unigene or a transcript between comparative groups was greater than one, and the p.adjust < 0.05 (Wald test), they were considered to be differentially expressed. GO function and KEGG pathway enrichment were performed by clusterProfiler (R package) [74] in each comparative group, using the p.adjust (Fisher’s exact test) of 0.05 as the cutoff for significance. P.adjust used in the study refers to the *p*-value adjusted by false discovery rate (FDR) controlled by BH procedure [75].

### qRT-PCR validation

The sequences of the candidate genes used for quantitative reverse transcription PCR (qRT-PCR) validation were extracted from the unigene assembly. Forward and reverse primers were designed using Primer3 [76] with the following parameters: Primer Size ranging from 17 to 23, Primer Tm ranging from 57.0 °C to 63.0 °C, Primer GC% ranging from 20.0 to 80.0, product size ranging from 100 to 300 (Additional file 2 Table S9). Primers were synthesized by TIANYI HUIYUAN, Guangzhou, China. RNA of the 18 samples were reverse transcribed to cDNA with PrimeScript RT Reagent Kit (Perfect Real Time) following the official manual. qRT-PCR was performed on LightCycler® 96 System using TB Green Premix Ex Taq II (Tli RNase H Plus) with the following program: 30 s preincubation at 95 °C, 40 cycles of 10 s denaturation at 95 °C, 20 s annealing at 56 °C, 15 s extension at 72 °C, one cycle of melting and cooling at 37 °C. The relative expression levels were calculated using the 2^−∆∆CT^ method [77], using housekeeping gene 18S rRNA as the internal standard. The calculated relative expression levels of each gene in each sample were shown in the bar plot with error bars, and the corresponding expression level calculated with transcriptome data were also shown in the plot for comparison (Fig. 5).

## Supplementary Information

**Additional file 1: Fig. S1**. Length distribution of the unigenes. **Fig. S2**. Expression level distribution of the unigenes. **Fig. S3**. Correlations between samples. **Fig. S4**. Clustering of samples. **Fig. S5**. Venn plot of the expressed genes in each tissues. The overlapping regions represent common expressed genes in the same tissue of different individuals. **Fig. S6**. Number of DEGs in each COG category. **Fig. S7**. Number of DEGs in the corresponding GO terms. **Fig. S8**. Significantly enriched KEGG pathways of DEGs of gill. **Fig. S9**. Significantly enriched KEGG pathways of DEGs of mantle. **Fig. S10**. Significantly enriched KEGG pathways of DEGs of adductor muscle.

**Additional file 2: Table S1**. Pairwise sequence identity and genetic distance of the concatenated dataset (atp6 + COI) between individuals used in the study and other B. spp. **Table S2**. Summary of transcriptome data before and after quality control. **Table S3**. Mapping rates. **Table S4**. Summary of unigenes and transcripts. **Table S5**. DEGs in the inter-individual comparision groups with the highest fold changes. **Table S6**. Annotations from each database. **Table S7**. Expression level matrix of genes in the line charts of Fig. 4. **Table S8**. Unigenes related to stress response expressed predominantly in the gills. **Table S9**. Primers of the candidate genes used in qRT-PCR validation.

## Data Availability

The RNA-sequencing data supporting the conclusions of this article is available through the BioProject accession number PRJNA740124 on NCBI SRA (Sequence Read Archive). Data not shown in the main text was included in Additional file 1 (Supplementary Fig. 1 - Supplementary Fig. 10) and Additional file 2 (Supplementary Table 1 - Supplementary Table 9).

## Abbreviations

O_GR: The gill of the younger individual
O_MT: The mantle of the younger individual
O_AM: The adductor muscle of the younger individual
Y_GR: The gill of the younger individual
Y_MT: The mantle of the younger individual
Y_AM: The adductor muscle of the younger individual
GR: Comparison between gills of the older and younger individual
MT: Comparison between mantle of the older and younger individual
AM: Comparison between adductor muscle of the older and younger individual
DEGs: Differentially expressed genes
qRT-PCR: Quantitative reverse transcription PCR
SLC: Solute carrier
TLR: Toll-like receptor
CLR: C-type lectin receptor
PGRP: Peptidoglycan recognition protein
PRR: Pattern recognition receptor
PAMP: Pathogen-associated molecular pattern
GIMAP: GTPase of the immune-associated protein
IIGP5: Interferon-inducible GTPase5
CLEC: C-type lectin
RAB5: Ras-related protein 5
EEA1: Early endosome antigen 1
RAC1: Ras-related C3 botulinum toxin substrate 1
PAK1: p21-activated kinase 1
CTBP1: C-terminal-binding protein 1
M6PR: Mannose-6-phosphate receptor
AP-3: Adaptor protein-3
VAMP7: Vesicle-associated membrane protein 7
VHA: Vacuolar-type H^+^-ATPase
UPS: Ubiquitin-proteasome system
ATG: Autophagy-related gene
DRAM: Damage-regulated autophagy modulator
SUOX: Sulfite oxidase
TST: Thiosulfate sulfurtransferase
SAT: Sulfate anion transporter 1
ROS: Reactive oxygen species
SOD: Superoxide dismutase
CAT: Catalase
GPX: Glutathione peroxidase
ABC: ATP-binding cassette
CASP: Caspase
BLOC1S1: Biogenesis of lysosome-related organelles complex 1 subunit 1
*atp6*: ATP Synthase Membrane Subunit 6
COI: Cytochrome c oxidase subunit I
