# Supplementary material for: Gene expression profiles provide insights into the survival strategies in deep-sea mussel (Bathymodiolus platifrons) of different developmental stages
Source: BMC Genomics. 2022 Apr 19;23(Suppl 1):311. doi: 10.1186/s12864-022-08505-9 (PMC9016928; doi:10.1186/s12864-022-08505-9)
Supplement: Supplementary file 1 — Additional file 1: Fig. S1. Length distribution of the unigenes. Fig. S2. Expression level distribution of the unigenes. Fig. S3. Correlations between samples. Fig. S4. Clustering of samples. Fig. S5. Venn plot of the expressed genes in each tissues. The overlapping regions represent common expressed genes in the same tissue of different individuals. Fig. S6. Number of DEGs in each COG category. Fig. S7. Number of DEGs in the corresponding GO terms. Fig. S8. Significantly enriched KEGG pathways of DEGs of gill. Fig. S9. Significantly enriched KEGG pathways of DEGs of mantle. Fig. S10. Significantly enriched KEGG pathways of DEGs of adductor muscle. [file 12864_2022_8505_MOESM1_ESM.docx]

**Additional File 1
(Supplementary figure 1 to Supplementary figure 10)**


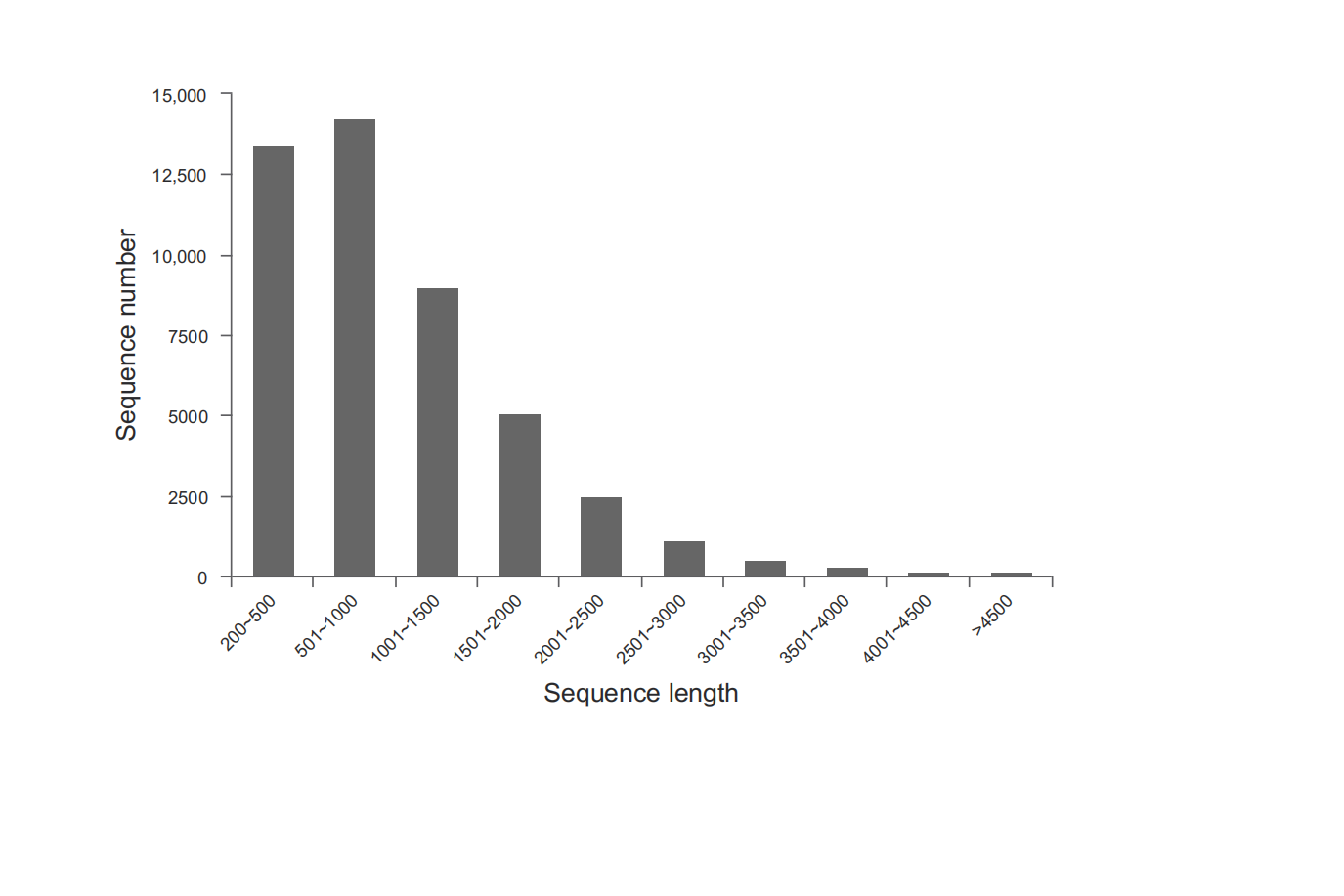


**Fig. S1** Length distribution of the unigenes.


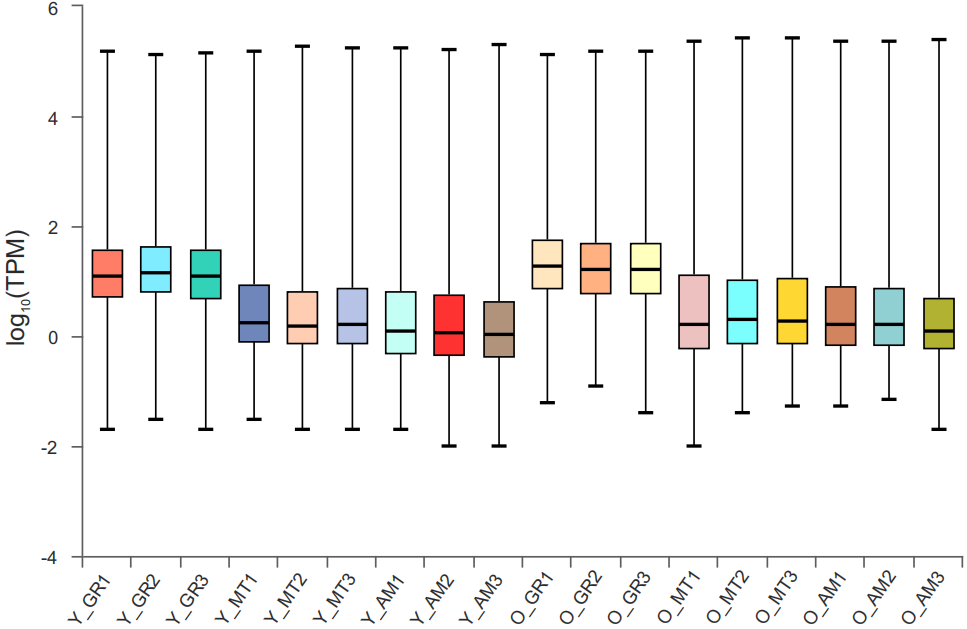


**Fig. S2** Expression level distribution of the unigenes.


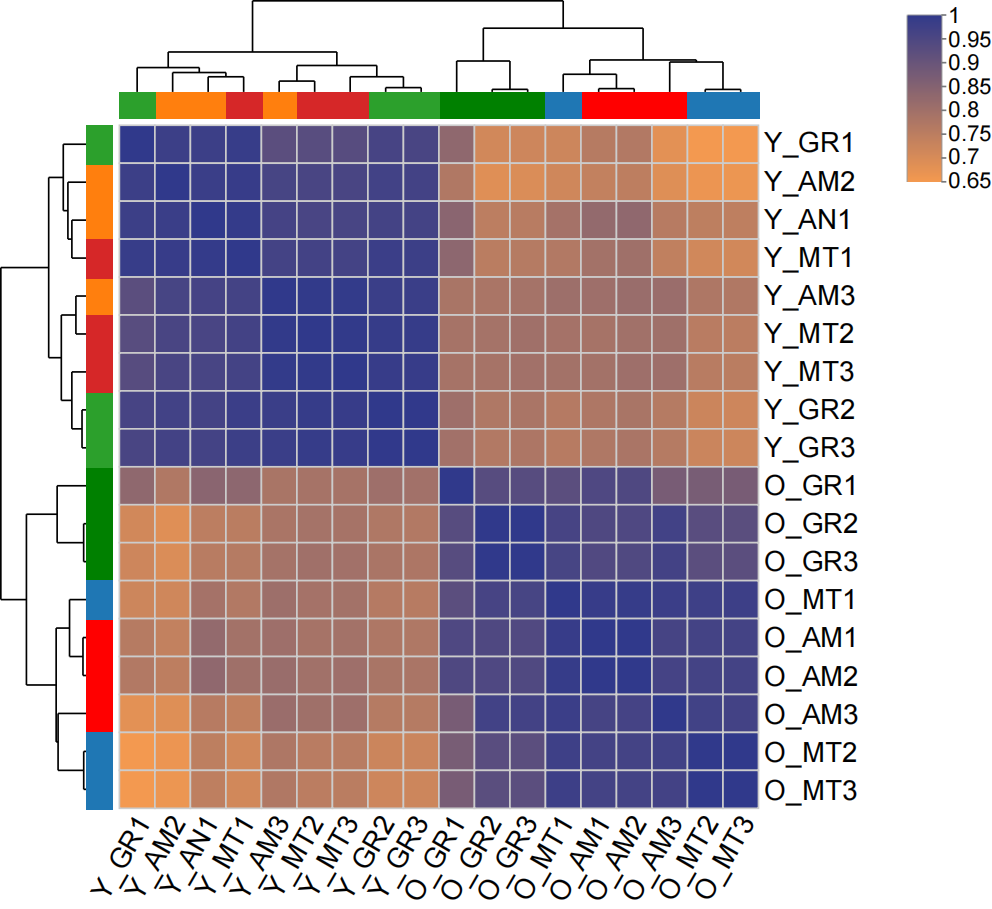


**Fig. S3** Correlations between samples.


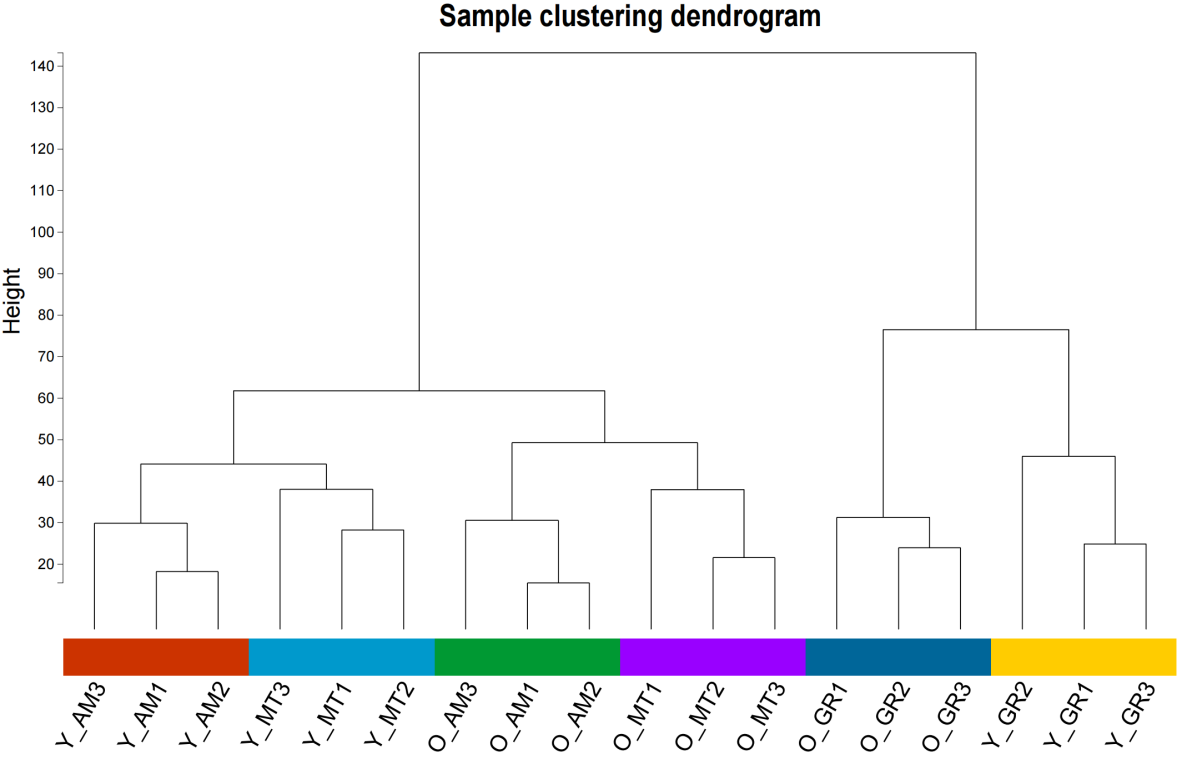


**Fig. S4** Clustering of samples.


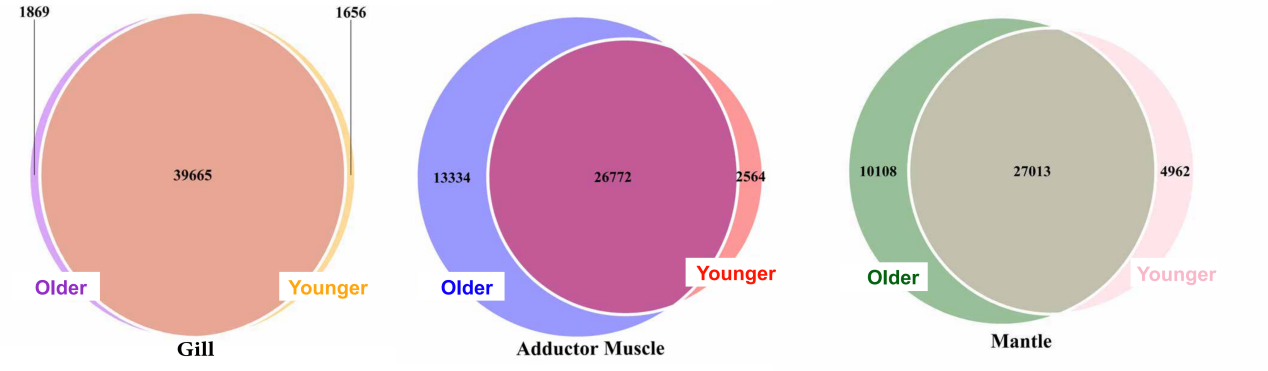


**Fig. S5** Venn plot of the expressed genes in each tissues. The overlapping regions represent common expressed genes in the same tissue of different individuals.


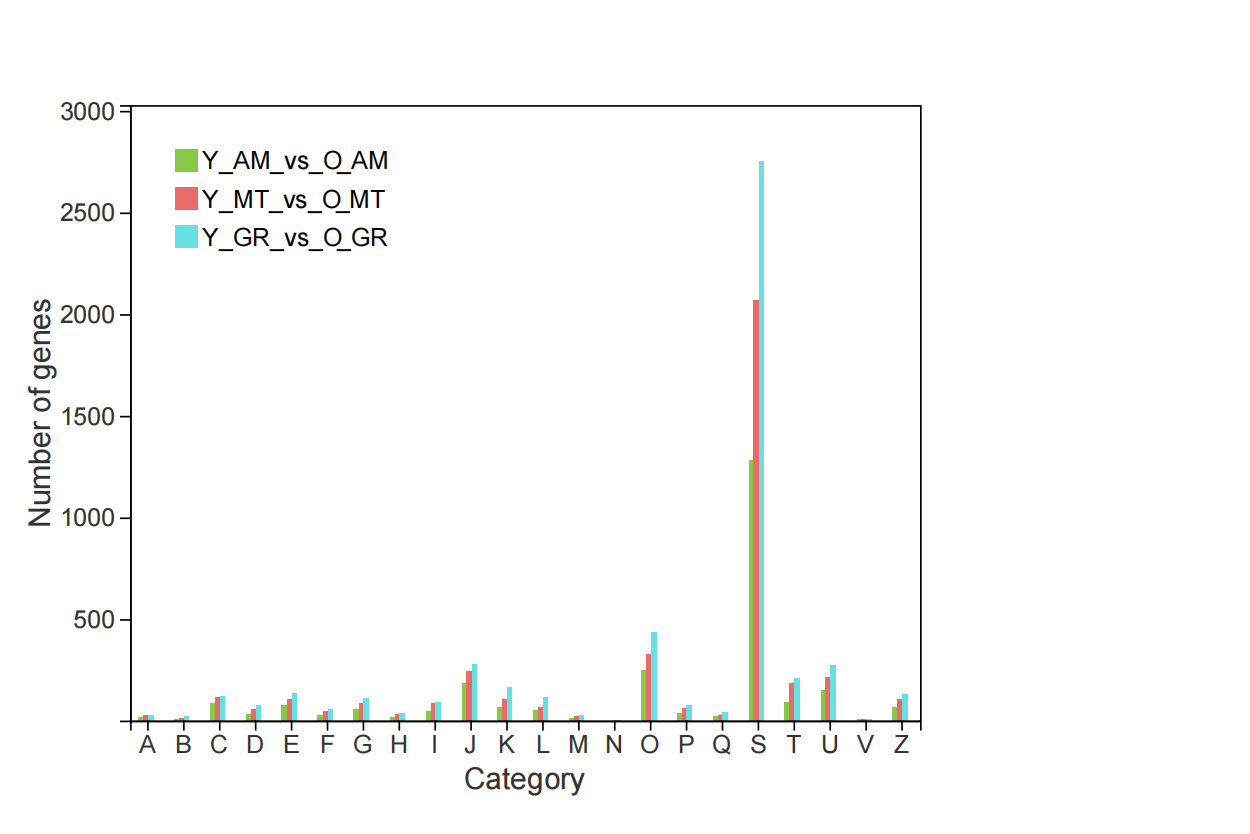


**Fig. S6** Number of DEGs in each COG category.


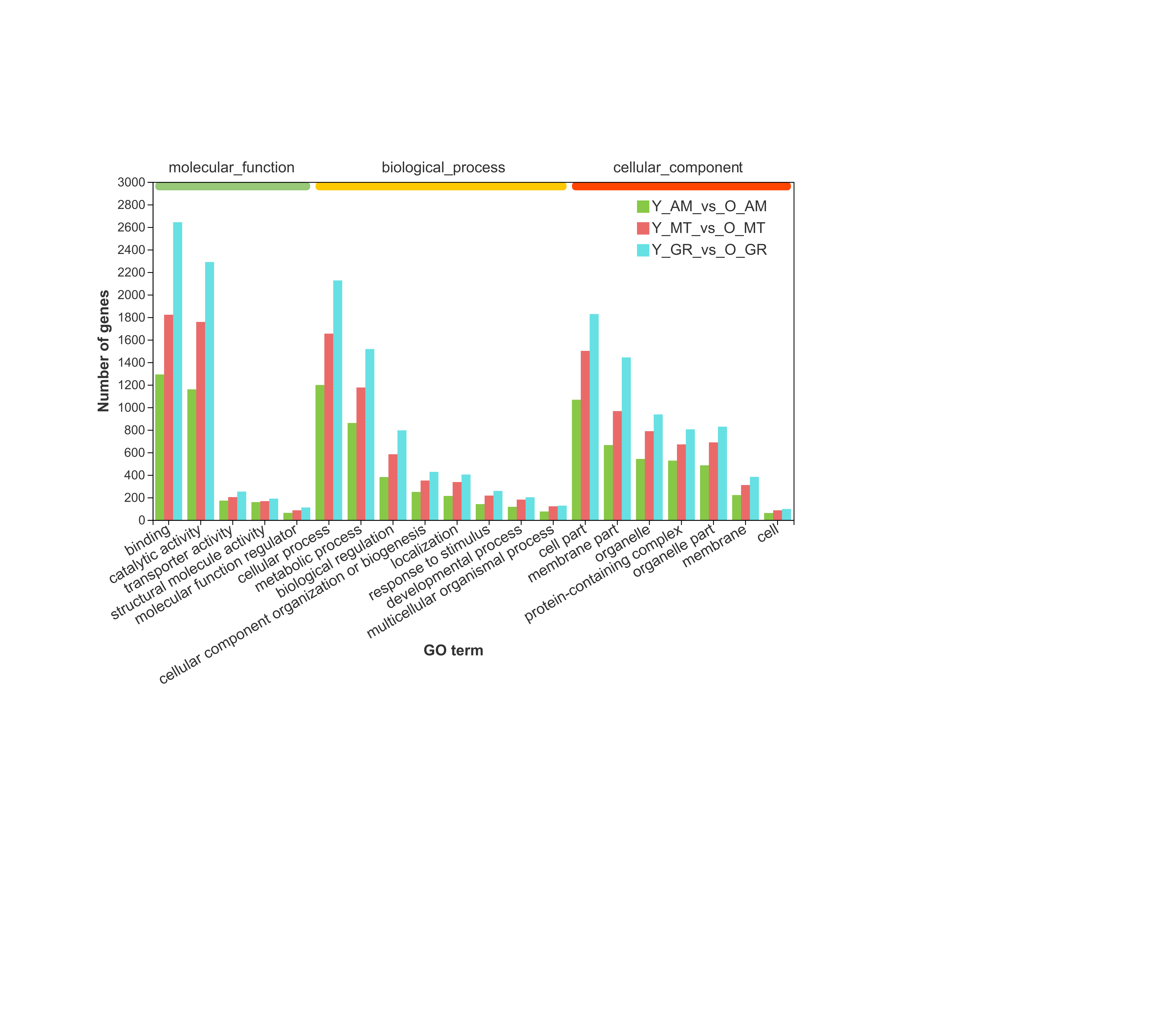
**Fig. S7** Number of DEGs in the corresponding GO terms.


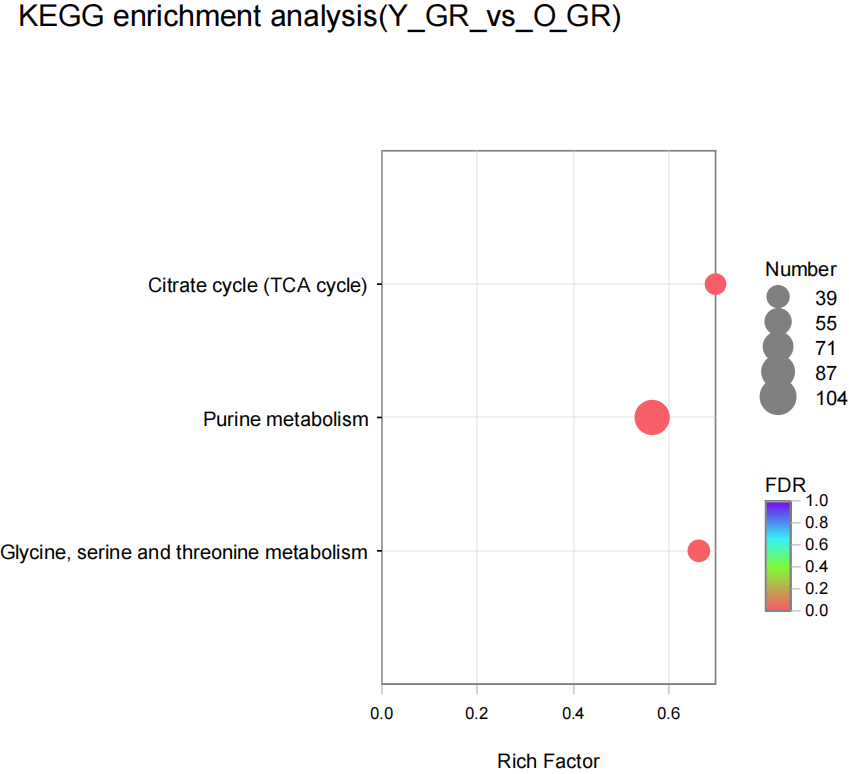


**Fig. S8** Significantly enriched KEGG pathways of DEGs of gill.


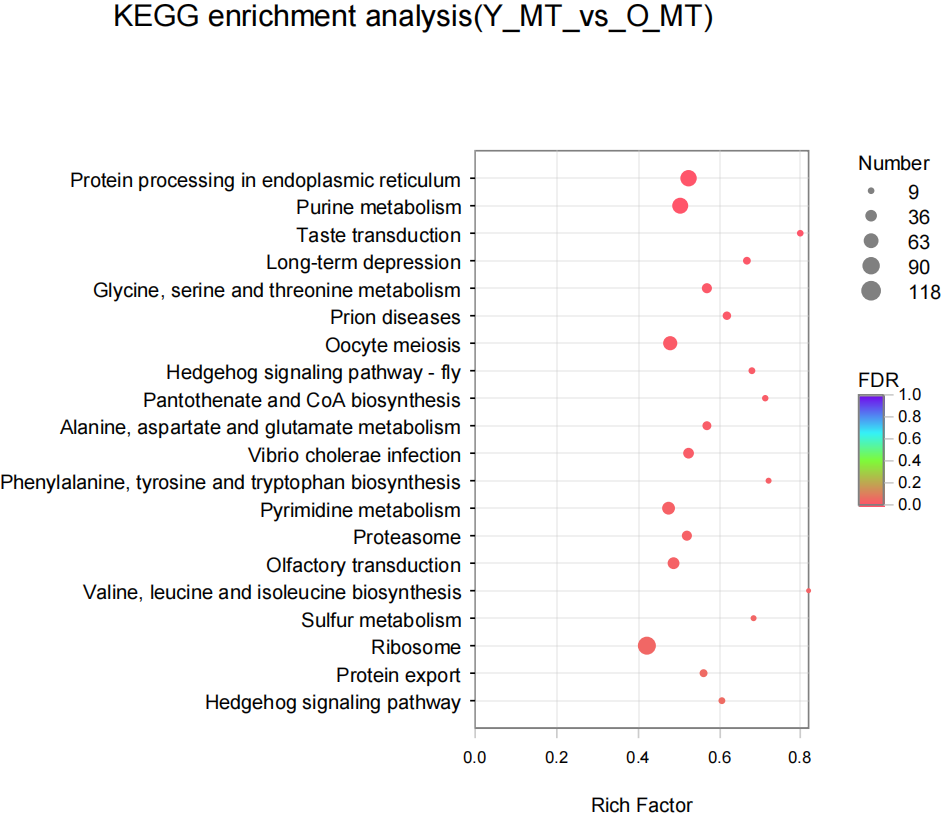


**Fig. S9** Significantly enriched KEGG pathways of DEGs of mantle.


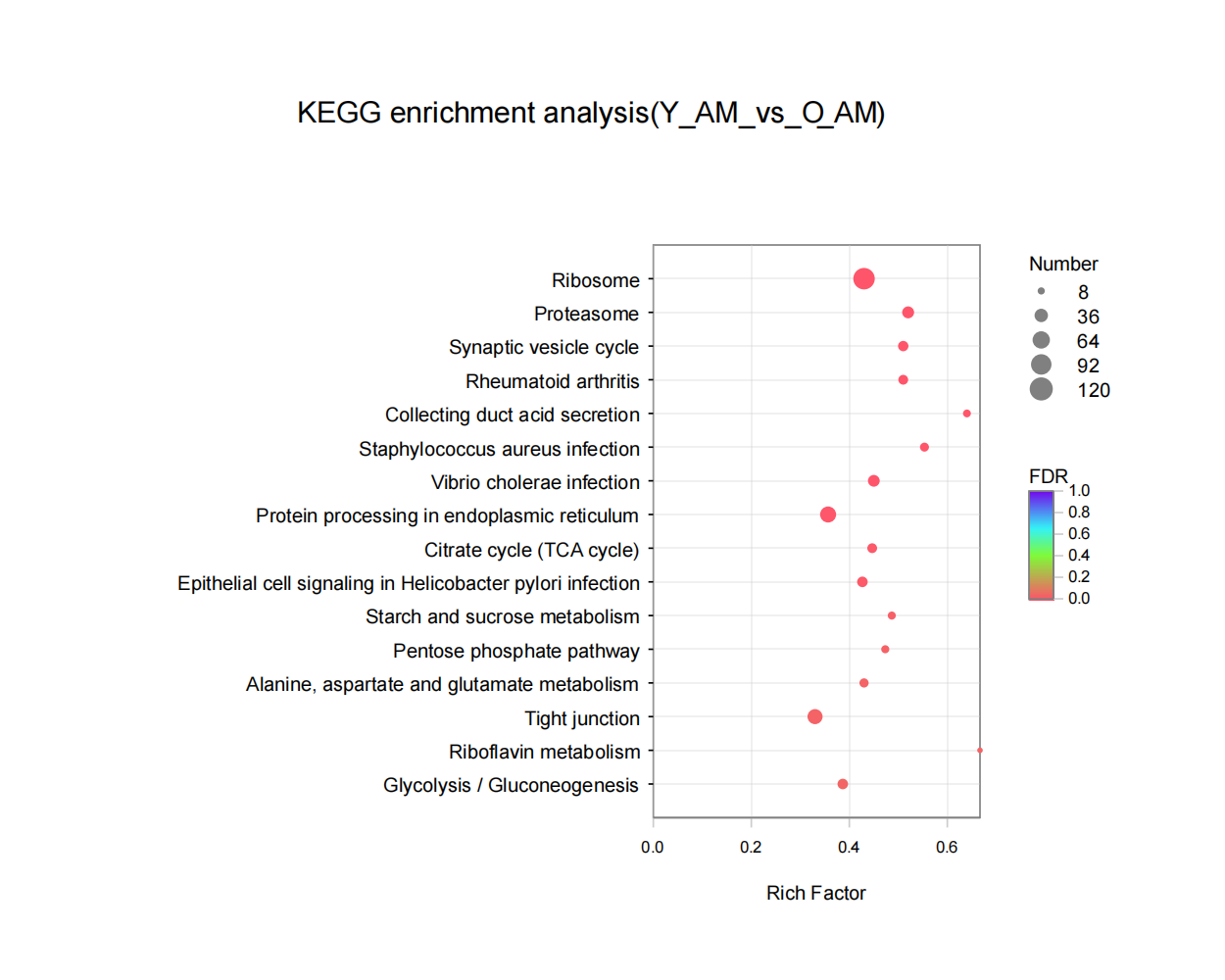


**Fig. S10** Significantly enriched KEGG pathways of DEGs of adductor muscle.
